# Regulatory Nucleotide Sequence Signals for Expression of the Genes Encoding Ribosomal Proteins

**DOI:** 10.3389/fgene.2020.00501

**Published:** 2020-06-05

**Authors:** Jihye Ryu, Chaeyoung Lee

**Affiliations:** School of Systems Biomedical Science, Soongsil University, Seoul, South Korea

**Keywords:** ribosomal protein, expression regulatory quantitative trait loci, *trans*-acting regulation, pQTL, lncRNA

## Abstract

Ribosomal proteins (RPs) are essential components that translate genetic information from mRNA templates into proteins. Their expressional dysregulation adversely affects the survival and growth of human cells. Nevertheless, little is known about the nucleotide sequences regulating the expression of RPs. Genome-wide associations for expression level of 70 RP genes were conducted across expression stages. Eighteen expression regulatory quantitative trait loci (erQTLs) were identified for protein abundances of 21 RPs (*P* < 1 × 10^–5^), but not for their mRNA expression and ribosome occupancy (*P* > 1 × 10^–5^). These erQTLs for protein abundance (pQTLs) were all *trans*-acting. Three of the pQTLs were associated with the expression of long noncoding RNAs (lncRNAs). Target genes of these lncRNAs may produce ribosomal components or may control the metabolic cues for ribosome synthesis. mRNAs of the RP genes extensively interact with miRNAs. The protein-specific erQTLs may become engendered by intensive miRNA controls at the translational stage, which in turn can produce RPs efficient in handling instantaneous cell requirements. This study suggests that the expression levels of RPs may be greatly influenced by *trans*-acting regulation, presumably via interference of miRNAs and target genes of lncRNAs. Further studies are warranted to examine the molecular functions of pQTLs presented in this study and to understand the underlying regulatory mechanisms of gene expression of RPs.

## Introduction

Ribosome is a complex organelle required for translating the genetic information of mRNA templates into proteins, and ribosomal proteins (RPs) are primary components of the ribosome in conjunction with ribosomal RNAs (rRNAs). In humans, 80 RPs are assembled into a ribosome together with four rRNAs (Anger et al., 2013), and over 200 factors are involved in the assembly (Thomson et al., 2013). Thus, expression of the RP genes should be exquisitely regulated throughout all stages of gene expression, considering that the RP genes are scattered over the entire human genome. For example, mammalian target of rapamycin (mTOR) signaling controls ribosome biogenesis via expressional regulation of RPs and rRNAs. Under sufficient nutrient condition and presence of growth factors, mTOR signaling increased both transcription and translation of RP genes (Mayer and Grummt, 2006). During transcription, mTOR-dependent transcription factors (e.g., SFP1 and FHL1) bind to the promoters of RP genes (Jorgensen et al., 2004). Moreover, mTOR signaling can activate chromatin state of the RP genes by recruiting histone acetyltransferase ESA1 (Reid et al., 2000), and phosphorylate p70 RP S6 kinase 1 (S6K1; Holz et al., 2005). In particular, S6K1 is a well-known coordinator of ribosome biogenesis and promotes transcription of numerous genes encoding nucleolar factors for ribosome biosynthesis such as rRNA synthesis and RP assembly/transport (Gao and Roux, 2015). During translation, the mTOR signaling promotes translation of the mRNAs with a 5′-terminal oligopyrimidine (5′TOP) sequence (Gentilella and Thomas, 2012). All RP genes include the 5′TOP sequence at their transcriptional start sites (Gentilella and Thomas, 2012), thereby leading to the coordination of ribosome biogenesis regulation in a cell.

For ribosome biogenesis, the cells control RP gene expression, thus ensuring stoichiometric production of RPs (Gupta and Warner, 2014). In general, certain RPs are excessively expressed beyond the cell requirement, and those that fail to assemble into ribosomal subunits are rapidly degraded by the nuclear ubiquitin–proteasome system; however, dysregulation of RP expression or perturbation of ribosome biosynthesis can lead to accumulation of unassembled RPs in the insoluble aggregates in the nucleolus (Sung et al., 2016). Such aggregation can provoke nucleolar stress and activate the MDM2–p53 tumor suppress pathway (Deisenroth and Zhang, 2010).

Although regulating the gene expression of RPs is crucial in maintaining cell growth and survival, the mechanism by which genetic factors regulate their gene expression remains unclear. The present study aimed to identify the expression regulatory quantitative trait loci (erQTLs) for RPs across expressional stages.

## Materials and Methods

### Expression Data

This study examined erQTLs for expression of RP genes at three different stages: mRNA transcription, ribosome occupancy, and protein abundance. We used expression data obtained from studies that assessed three kinds of expression levels using lymphoblastoid cell lines (LCLs) derived from unrelated Yoruba individuals of Ibadan in Nigeria (Pickrell et al., 2010; Battle et al., 2015). The mRNA transcript levels of 75 individuals were obtained by polyadenylated fractions of RNAs using the Illumina GA2 platform (Pickrell et al., 2010). The quantity was estimated with the median of 8.6 M reads per individual uniquely mapped to the Ensemble genes. Read counts were normalized by the total number of mapped reads and gene length and were recorded as reads per kilobase per million mapped reads (RPKM) of each gene for every individual. The ribosome occupancy levels of 72 individuals were produced by sequencing ribosome-protected fragments (RPFs) of mRNA isolated using Illumina HiSeq 2500 with ARTseq Ribosome Profiling kit (Epicenter, RPHMR12126; Battle et al., 2015). The median was 12.1 M reads per individual, and the read counts were also normalized. Protein abundance levels were obtained as ratios to the stable isotope labeling by amino acids in cell culture (SILAC) internal standard sample for the gene using protein mass spectrometry (Battle et al., 2015). Individuals lacking SILAC internal standard sample were excluded.

Expression regulatory quantitative trait loci were identified with the data centered and scaled to mean 0 and variance 1 and were then quantile-normalized to fit a standardized normal distribution. A principal component (PC) analysis was conducted to eliminate the unmeasured potential confounding effects. The PC count was determined by maximizing the number of identified erQTLs for each of three kinds of expression data. Six PCs were regressed out from mRNA expression, nine PCs from ribosomal occupancy, and seven PCs from protein abundance. Expression data of RPs used in the present study included mRNA expression of 70 RP genes (43 for large subunit and 27 for small subunit), ribosome occupancy of 69 RP genes (42 and 27), and protein abundance of 66 RP genes (39 and 27).

### Genotype Data

We used autosomal genotypic data of Yoruba individuals in a variant calling format produced from the 1000 Genomes Project Phase 3^1^. The sequence variants were excluded following the criteria of Hardy–Weinberg disequilibrium (*P* < 1 × 10^–6^) and the minor allele frequency (MAF) < 0.1. After the quality control, genotypic data for 5,594,467 nucleotide variants on 64 YRI individuals were retained for analyzing the erQTL association. Only individuals with both genotype and expression data were used for the present erQTL analysis. As a result, 63 individuals for eQTL analysis, 62 for rQTL, and 51 for pQTL were included.

### Additional Data

We further investigated erQTLs using various expression data to specify the regulatory mechanism of identified erQTLs. Initially, we identified cis-eGenes to understand the potential regulatory mechanism of trans-erQTL. The cis-eGene was defined as a protein-coding or noncoding gene within 1 Mb from erQTL. The erQTLs for protein-coding genes and lncRNAs were determined by transcriptomic data resulting from the Genotype-Tissue Expression Consortium (GTEx)^2^. The erQTLs for miRNA were examined for their expression in LCL of Yoruba individuals from the 1000 Genomes project (Lappalainen et al., 2013).

To discover the putative mRNA targets of lncRNAs identified as cis-eGenes, the lncRNA–RNA interaction was predicted based on the energy of intermolecular base-pairing interactions. These predictions were made with a cutoff value of 16 kcal/mol reduction in the interaction energy using Rtools^3^ (Terai et al., 2016).

The human miRNA interactome data produced by cross-linking, ligation, and sequencing of hybrids (CLASH) were used to evaluate the posttranscriptional regulation of RP genes. A total of 18,514 miRNA–target RNA interactions were available as chimeric sequencing reads between 6,959 genes and 399 miRNAs (Helwak et al., 2013). All the miRNAs paired with human RP genes were selected to examine whether miRNA–RP RNA interactions were enriched.

### Statistical Analysis

The erQTLs for RPs at mRNA transcription, ribosome occupancy, and protein abundance were identified using a mixed linear model. The analytical model for genome-wide erQTL included polygenic effects with a genomic similarity matrix (GSM) to avoid population stratification (Lee, 2018) as follows:

y=μ⁢1+X⁢β+g+ε

where ***y*** is the vector of expression levels for RP genes, μ is the overall mean, **1** is the vector of 1′s, β presents the fixed effects for the minor allele of the nucleotide variant to be tested for association, and ***X*** is the design matrix with elements of 0, 1, and 2 for the homozygote of the major allele, heterozygote of the minor allele, and homozygote of the minor allele, respectively. Vector ***g*** presents the random polygenic effects with $\text{g}∼N\,\left(0,\text{A}\,\sigma_{g}^{2}\right)$, where ***A*** is the GSM with elements of pairwise genomic similarity coefficients estimated using sequence variants and $\sigma_{g}^{2}$ is the polygenic variance component. The genomic similarity coefficient between individuals *j* and *k* can be calculated (Yang et al., 2011) as follows:

$$g_{j\,k}=\frac{1}{n_{\nu }}\,\sum_{i=1}^{n_{\nu }}\frac{\left(\tau_{i\,j}-2\,f_{i}\right)\,\left(\tau_{i\,k}-2\,f_{i}\right)}{2\,f_{i}\,\left(1-f_{i}\right)}$$

where n*_ν_* is the number of nucleotide variants that contribute to the genomic similarity, *τ_*ij*_*, and *τ_*ik*_* represent the number (0, 1, or 2) of minor alleles for the nucleotide variant *i*, and *f*_*i*_ is the frequency of the minor allele. Vector ε is the random residuals with $\varepsilon ∼N\,\left(0,\text{I}\,\sigma_{\varepsilon }^{2}\right)$, where $\sigma_{\varepsilon }^{2}$ is the environmental variance component and ***I*** is the identity matrix. The variance components for polygenic effects and residuals were estimated using restricted maximum likelihood (REML). The REML estimates were initially obtained by expectation–maximization algorithm and were then used as initial values to obtain their average information-based REML estimates. All the association analyses for erQTL were carried out using GCTA (v1.26)^4^. For genome-wide identification of erQLT, multiple testing was applied with a conservative significance threshold of *P* = 1.0 × 10^–5^ using permutation. The erQTL identification process was repeated for the three types of levels across the expression stages.

## Results

**FIGURE 1:**
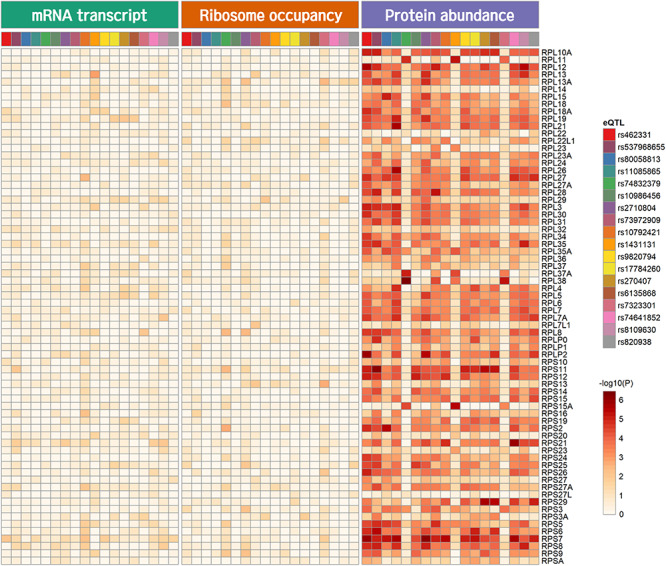
Heatmap of pQTL significance for ribosomal protein genes across expression stages. Significance of association with representative nucleotide variant at every pQTL signal is presented.

Genome-wide analysis of erQTL for RPs revealed associations of 132 nucleotide variants with protein abundance (*P* < 1.0 × 10^–5^), but any associations were not found with mRNA expression or with ribosome occupancy (*P* > 1.0 × 10^–5^). Linkage analysis showed that the 132 variants were included in 18 independent linkage disequilibrium blocks. The 18 pQTLs are presented with a representative variant at each association signal in Table 1. The signals except for rs10986456, rs10792421, and rs7323301 were shared by two or more RPs. Significances for the pQTLs across 21 RPs are presented in Figure 1. None of the 18 pQTLs was located within the corresponding eGene. Nine intragenic pQTLs were found within genes encoding five proteins (CACNA1A, TGFBR2, ATP11B, BMP6, and KIR2DS4) and four lncRNAs (RP11-483P21.3, AC083864.3, RP4-705D16.3, and AC07371.1). Five pQTLs in the protein-coding genes were not associated with expression of the corresponding gene or genes in the same chromosome regardless of their expressional stages (*P* > 0.05). In contrast, two pQTLs (rs462331 and rs2710804) in lncRNA genes were associated with the corresponding lncRNAs (RP11-483P21.3 and AC083864.3, respectively) in numerous human tissues (*P* < 1.0 × 10^–4^, Table 2). Moreover, rs462331 and rs10986456 were associated with their nearby lncRNAs, RP11-483P21.2 and RP3-377H17.2, respectively.

**TABLE 1:** Genome-wide association of genetic variants with abundance of ribosomal protein*.

pQTL*	**Position^**§**^**	**Allele^**¶**^**	**MAF**	**Gene^**‡**^**	**RP^**†**^**
rs462331	16:83832299	A/T	0.098	RP11-483P21.3	RPLP2, RPS7, RPL12, RPS26, RPL10A
rs537968655	4:117607965	A/G	0.167	–	RPS11, RPS6, RPS7, RPL10A, RPL28
rs80058813	4:117610565	G/A	0.225	–	RPS2, RPS7, RPL15
rs11085865	19:13680104	C/T	0.137	CACNA1A	RPL21, RPS7, RPL26, RPL3
rs74832379	4:34551579	T/A	0.147	–	RPL38, RPL37A
rs10986456	9:27602577	C/T	0.069	WDR38	RPS12
rs2710804	7:36084529	T/C	0.108	AC083864.3	RPS7, RPLP2, RPS8
rs73972909	2:154234327	T/A	0.127	AC079150.3	RPL28, RPS7, RPL12
rs10792421	11:63605177	G/A	0.147	MARK2	RPS7
rs1431131	3:30675880	A/T	0.225	TGFBR2	RPS15A, RPL11
rs9820794	3:182633827	T/G	0.118	ATP11B	RPS11, RPS7
rs17784260	12:4984022	T/C	0.137	KCNA6	RPS7, RPS6
rs270407	6:7738392	T/C	0.167	BMP6	RPS29, RPS11
rs6135868	20:16706651	A/C	0.127	RP4-705D16.3	RPS29, RPS11
rs7323301	13:27336447	C/A	0.147	GPR12	RPL38
rs74641852	21:21060812	G/A	0.108	–	RPS21, RPS7
rs8109630	19:55356516	A/C	0.127	KIR2DS4	RPL12, RPS8
rs820938	7:109282537	G/T	0.147	AC073071.1	RPS7, RPS29

*No erQTLs were found for mRNA expression or for ribosome occupancy (P > 1.0 × 10^–5^). Representative nucleotide sequence variants for each erQTL signal are presented. ^§^The chromosomal position (Chr: bp) is obtained from the GRCh37 reference genome. ^‡^The gene is located within ± 100 kb from the pQTL. ^¶^Major/minor allele. ^†^Ribosomal proteins for their abundance association with pQTL are presented. pQTL, protein quantitative trait loci; MAF, minor allele frequency; and RPs, ribosomal proteins.

**TABLE 2:** Association of pQTL for ribosomal protein with mRNA expression of near gene (*cis*-eGene).

eQTL*	***cis*-eGene**	***P*-value**	**Tissue**
rs462331	RP11-483P21.2^†^	9.1 × 10^–8^	Mucosa of esophagus
rs462331	RP11-483P21.3^†^	2.2 × 10^–7^	Tibial artery
rs462331	RP11-483P21.3^†^	1.8 × 10^–5^	Sun exposed skin of lower leg
rs2710804	PP13004	6.3 × 10^–7^	Testis
rs2710804	AC083864.3^†^	1.3 × 10^–5^	Subcutaneous adipose
rs10792421	C11orf84	6.1 × 10^–7^	Sun exposed skin of lower leg
rs10792421	NAA40	7.6 × 10^–5^	Thyroid
rs17784260	RP3-377H17.2^†^	3.0 × 10^–5^	Brain cortex

*These eQTLs were identified for *cis*-eGenes in the following column as listed and were originally found as pQTLs for RP in the present study. ^†^Long non-coding RNA.

Furthermore, the top-ranked 200 target genes were analyzed for their mRNA interaction with each of the four lncRNAs associated with pQTLs. Protein abundance of nine of the target genes was associated (*P* < 0.05) with the pQTLs identified in the present study (Table 3). Two (DFFA and VKORC1L1) of these nine target genes for lncRNAs were associated with multiple pQTLs (rs462331 and rs2710804) which were previously identified for expression of lncRNAs (RP11-483P21.2 and AC083864.3, respectively).

**TABLE 3:** Association of pQTL with gene targeted by *cis*-eGene*.

**pQTL**	***cis*-eGene (lncRNA)**	**Gene targeted by *cis*-eGene^**§**^**	**Relevant function^**†**^**	***P*-value**
rs462331	RP11-483P21.2	PLEKHA2 (3′UTR)	None	3.40 × 10^–4^
		VKORC1L1 (3′UTR)	Oxidative stress	8.54 × 10^–3^
		DFFA (3′UTR)	Apoptosis	1.03 × 10^–2^
		RPL37 (3′UTR)	Producing RPs	1.87 × 10^–2^
		EXOSC2 (3′UTR)	Producing rRNAs	3.31 × 10^–2^
		DENND4C (3′UTR)	Energy level	3.59 × 10^–2^
rs17784260	RP3-377H17.2	NCL (CDS)	Producing rRNAs	5.53 × 10^–3^
rs2710804	AC083864.3	HBS1L (3′UTR)	Producing RPs	7.92 × 10^–3^
		DFFA (3′UTR)	Apoptosis	8.40 × 10^–3^
		VKORC1L1 (3′UTR)	Oxidative stress	1.17 × 10^–2^
		RPS3A (3′UTR)	Producing RPs	2.09 × 10^–2^

*Among the identified *cis*-eGenes, only genes encoding lncRNA were examined. ^§^Target location is presented in parentheses. ^†^Functions of the target genes for RP demand or synthesis. UTR, untranslated region; CDS, coding sequence.

Of the 80 known RP genes in human genome, RNAs transcribed from 76 genes were found to interact with miRNAs using CLASH (Table 4). Their proportion (0.95) is significantly larger (*P* < 0.01) than that (0.35) for other human genes. The interactions for every RP RNA and the RP RNA targeted by a miRNA occurred more frequently than those for other human genes (*P* < 0.05).

**TABLE 4:** Number and enrichment ratio of ribosomal protein genes interacting with miRNAs.

	**Count**				**Ratio**		
	**Genes (A)**	**Interactions with miRNA (B)**	**Interacting genes (C)**	**Interacting miRNA (D)**	**C/A**	**B/C**	**B/CD**
All genes*	19,901	18,514	6,959	399	0.35	2.66	0.0067
RP genes	80	554	76	146	0.95	7.29	0.0499

*The protein coding gene count was referred from GENCODE V25.

## Discussion

We identified 18 erQTLs for protein abundance of 21 RPs: 10 RPs in a small subunit and 11 RPs in a large subunit (*P* < 1.0 × 10^–5^). They were all protein-specific and *trans*-acting erQTLs. Three (rs462331, rs2710804, and rs10986456) of these pQTLs were associated with lncRNAs (RP11-483P21.3, AC083864.3, RP11-483P21.2, and RP3-377H17.2) in *cis*-acting. Some genes predicted as targets of the lncRNAs shared the pQTLs with RP genes. The target genes may trigger the RP expression. For example, RPS3A, RPL37, NCL, and EXOSC2 are known to be involved in ribosome synthesis (Mitchell et al., 1997; Ginisty et al., 1998; Fremerey et al., 2016). Furthermore, DFFA is implicated in apoptosis by provoking DNA fragmentation (Liu et al., 1997), and expression of VKORC1L1 is inflated by oxidative stress (Westhofen et al., 2011).

Since ribosomes are essential for most cellular activities such as cell survival, cell growth, and development in every living cell, the genes encoding RPs are well known and stably expressed (De Jonge et al., 2007). About 70% of cellular transcription is involved in ribosome biogenesis. Moreover, 50% of RNAPII transcription and 90% of mRNA splicing are allocated to transcription of RP genes (Li et al., 1999; Warner, 1999). About 30% translation in cells is processed for ribosome biogenesis (de la Cruz et al., 2018). Thus, the RP expression seems to be a high energy-demanding process and requires efficient regulation of the fluctuating cellular environment.

Certain strategies are applied for the rapid production of RPs according to the cell demand. RPs are excessively produced. In general, 25% of RPs in the protein level are spared to cope with the unexpectedly increased demand (Metzl-Raz et al., 2017); however, translation is a high-energy-consuming process for amino acid synthesis and polypeptide assembly. This requires approximately 50% of ATP consumption in the rapidly growing yeast cells (Warner, 1999). In contrast, transcription utilizes only 10% of the energy required for protein production (Liu et al., 2016). Thus, an instantaneous translation may be an energy-efficient model for prompt production of proteins. Indeed, RP mRNAs are abundantly stored as inactive messenger ribonucleoprotein (mRNP) particles in the cytoplasm of quiescent cells and poised for translation (Mayer and Grummt, 2006; Patursky-Polischuk et al., 2009). Only RP pQTLs were identified in the present study due to the intensive translational control of RPs; that is, instantaneous changes from repressed to active state are greatly attributed to translational regulations. In particular, it is notable that erQTL for ribosomal occupancy was not found in the current study, considering that ribosomal occupancy is, in general, largely correlated with protein abundance (Battle et al., 2015). A substantial control after initiation of translation is suspected. Polysomes for RP transcripts have larger ribosome density and larger tRNA adaptation than those for non-RP transcripts to cope with a heavy translation burden for cellular activity (Riba et al., 2019). Nevertheless, elongation of translation is slower for RP transcripts than for other transcripts (Riba et al., 2019). These results imply that expression of the RP genes is intensively regulated by controlling elongation speed of translation. The elongation speed can be controlled by frequent interaction of RP mRNA with miRNA. This was supported by the current study where various miRNAs were bound to RP mRNA as shown in Table 4. Numerous RPs (95%) were regulated by miRNAs, with three times more frequent interactions than other human genes. Moreover, RP genes were regulated 7.49 times more frequently by one miRNA on average than by other human genes. The substantial interactions with miRNA may efficiently regulate the RP expression. The fine-tuning in translational regulation by various miRNAs is crucial for ribosome heterogeneity (Genuth and Barna, 2018).

The 18 pQTLs found in the present study were all *trans*-acting pQTLs. Among these, 15 pQTLs were associated with multiple RPs. We initially tried to discover *cis*-eGenes of the *trans*-acting pQTLs. As a result, seven *cis*-eGenes were identified, and four of them were lncRNAs (Table 2). The lncRNAs were five times larger than those (0.8) that resulted from non-RP pQTLs (*P* < 0.05; Supplementary Table S1). In general, lncRNAs regulate the expression of their target genes after transcription via base-pairing interactions between lncRNA and RNA (Kretz et al., 2013). These lncRNAs may regulate the gene expression that may further control expression of RP genes. Protein abundance of nine target genes, which presumably interact with the four lncRNAs, is known to be associated with the pQTLs originally identified in the present study (Table 3). The target genes may contribute to ribosome synthesis by producing rRNAs or RPs itself or indirectly by controlling the metabolic cues that influence ribosome synthesis (Table 3). For example, RPL37 and RPS3A as the RPs were found to be regulated by lncRNA. HBS1L controls ribosome-associated mRNA surveillance, retrieving stalled ribosome during translation and maintaining ribosome homeostasis (Shoemaker et al., 2010). NCL and EXOSC2 produce rRNA (Mitchell et al., 1997; Ginisty et al., 1998; Fremerey et al., 2016). The rRNAs as major components for ribosome synthesis are expressed simultaneously with the RP genes. In particular, transcription of rRNAs strictly controlled the expression of RP genes in yeasts (Granneman and Tollervey, 2007). Moreover, NCL is known to play a pivotal role in ribosome assembly and biogenesis as well as in rRNA transcription and maturation (Ghisolfi et al., 1992; Bouvet et al., 1998). The NCL can control the expression of RPs by modulating the posttranscriptional expression of apoptosis-related genes such as BCL2, CCNI, and TP53 (Sengupta et al., 2004; Takagi et al., 2005; Abdelmohsen et al., 2011). Target genes with a function of regulating metabolic cues for ribosome synthesis include DFFA, VKORC1L1, and DENND4C. DFFA as an inhibitor of CAD is normally present in a complex with CAD. A cleavage between DFFA and CAD can provoke DNA fragmentation, a primary process of apoptosis (Sakahira et al., 1998). VKORC1L1 decreases the level of vitamins, K 2,3-epoxide, and K quinone. This reductase contributes to vitamin K-mediated protection against oxidative stress and conversion to active vitamin K (Westhofen et al., 2011; Karasawa et al., 2013). Thus, it plays a pivotal role in cell survival and apoptosis via intracellular antioxidation (Westhofen et al., 2011) and binding of the active vitamin K to BAK (Karasawa et al., 2013), respectively. DENND4C can facilitate glucose transportation into a cell as a guanine nucleotide exchange factor for Rab10 that is required for translocating GLUT4 (Sano et al., 2011). Cellular glucose is essential for promoting mTOR signaling (Dibble and Manning, 2013) and thereby affects the RP expression. Moreover, mTOR can be also facilitated by other metabolic cues such as apoptosis and oxidative stress. It triggers expression of various RPs by *trans*-pQTL. Such *trans*-regulations might considerably explain various protein levels in human cells. A previous pQTL study of human blood plasma revealed that multiple variants of a single gene can regulate even 60% of protein variance (Suhre et al., 2017). Knowledge regarding the underlying mechanisms of these *trans*-regulations is limited only to the regulation by few intermediates such as miRNA.

The present study identifies 18 erQTLs for RP genes in humans, and they are all pQTLs and *trans*-acting. Notably, the RP expression might be triggered by the target gene of lncRNA that is the very *cis*-eGene of the *trans*-acting pQTL. Moreover, RPs can be efficiently regulated via substantial interactions with miRNAs after the transcriptional stage. The erQTLs across expressional stages would help in understanding the underlying regulatory mechanisms in RP expression.

## Data Availability Statement

Publicly available datasets were analyzed in this study. This data can be found here: GSE61742.

## Author Contributions

JR and CL conceived the work, analyzed the data, interpreted the results, and wrote the manuscript.

## Conflict of Interest

The authors declare that the research was conducted in the absence of any commercial or financial relationships that could be construed as a potential conflict of interest.

## Footnotes

1 ftp://ftp.1000genomes.ebi.ac.uk/vol1/ftp/release/20130502/

2 https://gtexportal.org/

3 http://rtools.cbrc.jp/cgi-bin/RNARNA/index.pl

4 http://cnsgenomics.com/software/gcta/
