# Supplementary material for: Regulatory Nucleotide Sequence Signals for Expression of the Genes Encoding Ribosomal Proteins
Source: Front Genet. 2020 Jun 5;11:501. doi: 10.3389/fgene.2020.00501 (PMC7326009; doi:10.3389/fgene.2020.00501)
Supplement: Supplementary file 1 [file Data_Sheet_1.PDF]

Supplementary Material

Supplementary Table 1 Number of lncRNAs associated with randomly selected non-RP pQTLs

Random group	erQTL*	non-RP gene	cis-eGene	# of lncRNAs[¶]
Set 1	rs77181827	RCN1		1
	rs73516133	RTCA		
	rs221655	M6PR	POPDC3	
	rs59977050	AFG3L2		
	rs12457828	SH3GLB2	C18orf8, NPC1, TMEM241	
	rs923089	CUL2		
	rs6488666	NOP56	ARHGDIB	
	rs115781914	FAM98B	AGMO	
	rs77528738	ADK	PDZD8	
	rs77650776	ARHGAP30		
	rs7204658	NUP210		
	rs2123946	PRMT5	ZNF644	
	rs113492734	NHLRC2		
	rs7198241	CORO1C	SYCE1L,VN2R10P	
	rs12262944	COPS3		
	rs1551060	PSME1		
	rs2041998	PSMD14	AKR1B10	
	rs915223	TALDO1	LINC01653[†]	
Set 2	rs891158	ARL6IP5		0
	rs7186998	COPS3		
	rs138704378	ARHGAP30	x	
	rs190669981	USP10		
	rs2684409	ACADVL		
	rs112753101	PSMD13	BHLHE22	
	rs112602025	POLDIP3		
	rs62147135	USP10		
	rs4143638	NUP210		
	rs1791235	SCYL1	DSG2	
	rs9665943	LNPEP	FAM181B, RAB30-AS1	
	rs7525965	PPP6R3		
	rs16971178	IFIT1		
	rs6772323	MAD2L1	RP11-305K5.1, KCNAB1	
	rs73568952	COPS3		
	rs113873388	OARD1		
	rs114081983	PRMT3		
	rs1542856	RANBP3		
Set 3	rs73077082	PPP6R3		1
	rs1395787	RCN1	CEP97, NXPE3	
	rs2647445	COPS3	RAB3GAP2	
	rs73768411	APPL1		
	rs58500174	ADGRE5		
	rs73287156	SEC62		
	rs59156843	ARHGAP30	RP11-313F23.4[†]	
	rs75413058	CUL1		
	rs2385540	ERH		
	rs75252299	CTSH		
	rs112646586	NDUFB10		
	rs7947525	CBR4	APOA1, RP11-109L13.1	
	rs79714703	CBR1		
	rs73494415	VAMP8		

	rs12377254	HAT1		
	rs3848083	COPS3		
	rs13392364	CSNK2A2	WDSUB1	
	rs56862128	DTD1		
Set 4	rs10967945	HAT1		0
	rs9292230	YWHAH		
	rs72749968	RTCA		
	rs1487850	BAG6	SYT9	
	rs76141525	EHD4		
	rs7553391	POLDIP3		
	rs112972776	EXOSC1		
	rs9477979	POLDIP3		
	rs7202329	HEATR5B		
	rs28823013	CBR1	MPP5	
	rs7202299	HSD17B8		
	rs4581009	FBL		
	rs11750327	ENOPH1		
	rs2342734	PSMC3		
	rs7488361	TPI1	C1QL4	
	rs6490236	SERPINB9	SRRM4	
	rs1864807	RTCA		
	rs6443985	LGALS9		
Set 5	rs9444452	ACSL3		1
	rs61907585	PCCB	RP11-680H20.2[†], GPR83, C11orf54, HEPHL1	
	rs79017261	RCN1		
	rs1580409	ADK		
	rs587889	NUP160	TPT1P4	
	rs8056203	UBE2L6		
	rs9728262	OAS3		
	rs9271884	TRAPPC1	HLA-DRB5	
	rs1552981	SLC25A19		
	rs386077	NHLRC2		
	rs9292337	IGF2BP3		
	rs1697982	COPS3	CTD-2589H19.6, SLC9A3, CEP72	
	rs115023906	ARHGAP30		
	rs17045478	ACADVL		
	rs2577146	COPS3	RAB3GAP2	
	rs58281172	CD53		
	rs11067015	COX5B		
	rs12977081	PSMA2		
Set 6	rs1828092	MYO1E	PTCHD4	0
	rs138478263	MRPL50		
	rs112972776	EXOSC1		
	rs60478584	VPS35L		
	rs13234737	COPS3		
	rs113759463	CSNK2A2		
	rs7202299	HSD17B8		
	rs60913694	FAM98B		
	rs1426409	LDAH	NWD2	
	rs58651349	SARS2	RFK, RPSAP9, GCNT1	
	rs10896977	SH3GLB2	STX3	
	rs1949106	RCN1		
	rs7761210	DNAJC13	HLA-E, ZFP57	
	rs74966096	AK2		
	rs75426602	ENY2		
	rs58863367	ARMT1	ATP5G3, HOXD10	
	rs75010541	IFIT1		
	rs8011039	PPP6R3	MAP4K5, SAV1, RP11-248J18.2, CDKL1	

Set 7	rs9292337	IGF2BP3		1
	rs73043693	FAM98B	SDK1	
	rs145851917	KPNA6		
	rs7128269	BAG6	SYT9	
	rs76723113	USP10		
	rs2891538	TPP1		
	rs73928418	SH3GLB2		
	rs11259205	GDI2		
	rs2362254	UBE2L6		
	rs1171572	VDAC1	NAXE, TTC24	
	rs78040874	ARL1	ZNF467	
	rs79408529	NCL		
	rs28378540	UGDH		
	rs62420792	OARD1	IL20RA	
	rs1648407	LDAH	NWD2	
	rs12487917	NUP210		
	rs75430989	AFG3L2		
	rs73584997	EEF2	CTD-2568A17.1†, ACPT, C19orf48	
Set 8	rs12465096	AFG3L2		2
	rs61413307	ATP5MD	ACADL	
	rs62174996	ANXA5		
	rs4048474	COPS3	PCNX1	
	rs77751273	GLO1		
	rs2891538	TPP1		
	rs7573728	COPS3	GPR155, SCRN3, CIR1	
	rs1795995	ACADVL		
	rs154065	ENOPH1		
	rs74051545	COPS3		
	rs57665111	PURA		
	rs17090982	PPP6R3		
	rs10977717	SNRPE		
	rs6916690	FAM98B		
	rs2102123	SCO1	RP4-660H19.1†	
	rs77271376	VDAC1		
	rs8095089	COPS3		
	rs59156843	ARHGAP30	RP11-313F23.4†	
Set 9	rs76094247	SHMT2		1
	rs6488666	NOP56	ARHGDIB	
	rs8057988	HSD17B8		
	rs74041569	SH3GLB2		
	rs2791795	CPSF2	GRIK2	
	rs116012439	CTSH		
	rs113365491	AK2	CHADL, EP300-AS1, RP4-756G23.5, TOB2	
	rs73952411	AAK1		
	rs16936109	ERH		
	rs72892054	PSPH	ACCS	
	rs6433532	ARMT1	ATP5G3,HOXD10	
	rs11067015	COX5B		
	rs76614247	FAM98B		
	rs77151223	SHMT2		
	rs10917830	TALDO1		
	rs7243764	DEF6	OSBPL1A, C18orf8	
	rs12096286	SCO1	RP4-660H19.1†	
	rs145816859	TKT		
Set 10	rs10129098	EIF2B5		1
	rs7125446	CBR4	SIK3	
	rs12646458	COPS3	RP11-707A18.1†	
	rs2834690	HM13		
	rs140603699	TKT		

rs10897452	IARS2	NAA40, C11orf84, COX8A, PLA2G16
rs57535018	AFG3L2	
rs74754604	NDUFB8	XIRP2, XIRP2-AS1, B3GALT1
rs2393277	IDE	
rs145167532	IVD	
rs76143456	CHUK	
rs10004662	UGDH	
rs7932915	COPS3	
rs62115414	MDH1	
rs5030317	DNAJC7	WR1-AS
rs12044584	COPS3	LYPD8, SH3BP5L, OR14I1
rs2401019	XPO1	
rs74992793	USP10	HTT

*The erQTLs are pQTLs associated with protein abundance of non-RP genes presented in the next column.

¶ The count of lncRNAs as a cis-eGene

† long non-coding RNA
